# Non-alcoholic fatty liver disease--a chronic disease of the 21^st^ century

**DOI:** 10.7555/JBR.31.20160153

**Published:** 2018-03-10

**Authors:** Peter Metrakos, Tommy Nilsson

**Affiliations:** Cancer Research Program, Block-E, The Research Institute of the McGill University Health Centre and Department of Medicine, McGill University, Montreal QC H4A 3J1, Canada.; Cancer Research Program, Block-E, The Research Institute of the McGill University Health Centre and Department of Medicine, McGill University, Montreal QC H4A 3J1, Canada.

**Keywords:** non-alcoholic fatty liver disease, pathogenesis, molecular mechanism, diagnostics, biomarker

## Abstract

Non-alcoholic fatty liver disease (NAFLD) comprises a spectrum of metabolic states ranging from simple steatosis to inflammation with associated fibrosis to cirrhosis. Though accumulation of hepatic fat is not associated with a significant increase in mortality rates, hepatic inflammation is, as this augments the risk of terminal liver disease, i.e., cirrhosis, hepatic decompensation (liver failure) and/or hepatocellular carcinoma. Disease progression is usually slow, over a decade or more and, for the most part, remains asymptomatic. Recent estimates suggest that the global prevalence of NAFLD is high, about one in four. In most cases, NAFLD overlaps with overweight, obesity, cardiovascular disease and the metabolic syndrome with numerous contributing parameters including a dysregulation of adipose tissue, insulin resistance, type 2 diabetes, changes in the gut microbiome, neuronal and hormonal dysregulation and metabolic stress. NAFLD is diagnosed incidentally, despite its high prevalence. Non-invasive imaging techniques have emerged, making it possible to determine degree of steatosis as well asfibrosis. Despite this, the benefit of routine diagnostics remains uncertain. A better understanding of the (molecular) pathogenesis of NAFLD is needed combined with long-term studies where benefits of treatment can be assessed to determine cost-benefit ratios. This review summarizes the current state of knowledge and possible areas of treatment.

## Introduction

Overweight and obesity are hallmarks of the metabolic syndrome and affect a large proportion of the general population. Of associated disease states, type 2 diabetes (T2D) and cardiovascular disease (CVD) show a marked increase in mortality rates. Non-alcoholic fatty liver disease (NAFLD), fat accumulation in the liver caused by factors other than alcohol, is a common manifestation of the metabolic syndrome yielding hypertriglyceridemia and abnormal hepatic fat accumulation presented either as simple steatosis (non-alcoholic fatty liver (NAFL)) or non-alcoholic steatohepatitis (NASH), the latter usually in conjunction with fibrosis. Patients with NASH/fibrosis are at risk of developing terminal liver disease through progressing to cirrhosis and hepatic decompensation requiring liver transplantation. Alternatively, NASH and fibrosis may progress to hepatocellular carcinoma (HCC). The typical time-frame of disease progression is a decade or more, i.e. NAFLD is best viewed as a chronic disease. During progression, NAFLD often remains asymptomatic and is usually diagnosed incidentally.

## Epidemiology of NAFLD and overlap with obesity, T2D and CVD

The prevalence of NAFL in the global population is around 25% and in the presence of obesity, as high as 51%. The overall global prevalence of NASH is estimated to be between 1.5%-6.45%^[1]^. There exists a significant overlap between NAFLD and NASH with obesity, 51% and 82%, respectively, and CVD with cardiac-related deaths as being one of the most common outcomes for NAFLD patients^[2-3]^. Indeed, the global prevalence of hyperlipidemia/dyslipidemia among NAFLD and NASH patients is estimated to be 70% whereas hypertriglyceridemia in NAFLD and NASH patients is estimated to be 40.7% and 83.3%, respectively^[1]^. In addition, hypertension has been diagnosed in 39.3% and 68% of NAFLD and NASH patients, respectively^[1]^. For patients with T2D, NAFLD was reported in 76% of the cases studied using acohort selected for normal rather than elevated plasma levels of aminotransferases^[4]^. About 56% of the patients of this cohort had NASH underscoring the apparent link between NAFLD/NASH and T2D as well as the limitations of using aminotransferases as the only means of diagnosing NAFLD (see below). In one study, the pooled overall diabetes prevalence in NAFLD and NASH patients were 22.5% and 43.6%, respectively^[1]^. Fibrosis progression in conjunction with NASH is seen in about half of NASH patients placing these at risk of developing terminal liver disease including HCC which has one- and three-year survival rates of 36% and 17%, respectively (for discussion on NAFLD and links to HCC, please see^[5-6]^). Resection, ablation and/or transplantation improves survival rates (one- and three-year survival rates of 70% and 55%, respectively)^[7]^ for patients diagnosed with HCC. In the case of hepatic decompensation, liver transplantation is the only viable option. Given the prevalence of NAFLD and NASH in the general population, the availability of suitable organ grafts becomes increasingly restricted necessitating research and method developments in the areas of *ex-vivo* reconditioning (e.g. defatting) of donor livers prior to transplantationas well as improving assessment of border-line grafts (extended criteria).Such improvements are feasible given that patients have already been transplanted with livers kept at 37°C *ex vivo* using a normothermic perfusion system^[8]^.

## NAFLD-NASH diagnostics

NAFLD is diagnosed as hepatic fat deposition (in cytoplasmic lipid droplets, CLDs) in more than 7% of the hepatocytes (as deduced by histochemistry). This usually occurs in the centrilobular zone 3 localized around central veins. Associated ballooning of hepatocytes signifies NASH, which is usually cryptogenic in nature. Whereas it is commonly agreed that NAFLD without NASH has slow or negligible histological progression, patients with NASH may exhibit progression to terminal liver disease (cirrhosis, HCC or decompensation). Apart from hepatic ballooning, NASH is often associated with an invasion of leukocytes and differentiation of hepatic stellate cells, a cell type that represents 5-8% of liver cells and store vitamin A deposited in CLDs. In NASH, activated hepatic stellate cells lose their vitamin A deposits, migrate to sites of damage (e.g. dead cells) and produce collagen, i.e., producing fibrinous tracks (revealed by tri-chrome stain). NASH is often seen together with fibrosis and, at some point, becomes irreversible with subsequent progressing to cirrhosis. All this takes time, sometimes several years to a decade. Meanwhile, liver conditions may remain asymptomatic. As such, NAFLD and NASH are usually diagnosed incidentally or in conjunction with one or more co-morbidity (e.g. obesity). As the prevalence of NAFLD is on the increase^[5-6]^ and overlaps with the metabolic syndrome, clinical practice guidelines now recommend that patients with obesity and/or T2D should be examined for NAFLD^[9]^. High triglyceride levels in combination with low serum HDL are also known to be common in patients with NAFLD and frequent (50%) in patients with dyslipidemia attending lipid clinics^[10]^. Taken together and given the observed prevalence of NAFLD, an argument for routine NAFLD diagnostics seems obvious. Such an argument, however, is countered by a high cost/benefit ratio coupled with an increased risk for the patient.

The diagnosis and staging of NAFLD has for a long time focused on histology-based evaluation of liver biopsies taken from liver grafts or patients suspected of having liver damage. This approach, especially with respect to staging of NASH, remains the gold-standard for diagnosis. Both percutaneous and transjugular liver biopsies, however, have associated complications and though difficult to pinpoint exactly, a non-negligible mortality rate. Complications include hemorrhage (0.35%-0.5%), puncture of other vicera (0.01%-0.1%) and moderate to severe pain (1.5%-3%) (see Guidelines on the use of Liver Biopsy in Clinical Practice by A. Grant, J. Neuberger, C. Day and S. Saxseena available at www.bsg.org.uk). A biopsy represents only a 1/50000^th^ of the liver and is therefore prone to significant sample error, i.e., that the biopsy does not accurately capture the disease state as steatosis, hepatitis and fibrosis are manifested unevenly^[11-12]^. There is also an apparent subjectivity in pathology-based assessment of NAFLD^[13]^. As such, liver biopsy is not considered suitable as part of routine diagnostics and is recommended only as a means of in-depth assessment of disease severity^[9]^.

Non-invasive diagnostics center around serum markers and imaging. AST-to-platelet ratio index (APRI) has an AUROC of 0.842 as determined in patients with hepatitis C virus^[14]^. BMI, hyperglycemia, platelet count and albumin are factors that are added to the AST/SLT ratio and is referred to as the NAFLD fibrosis score calculated according to published formula (www.nafldscore.com) (see also hepatic steatosis index, HIS, for comparison^[15]^). The NAFLD fibrosis score has an AUROC of 0.85 predicting advanced fibrosis (bridging fibrosis and cirrhosis) with a 90% sensitivity and 60% specificity to exclude (at a score of<κ-1.455) and a 67% sensitivity and 97% specificity to identify advanced fibrosis (at a score of>0.676). Another set, the enhanced liver fibrosis panel, examines the plasma levels of two matrix-turnover proteins (PIIINP and TIMP-1) and hyaluronic acid and has a AUROC of 0.90 with 80% sensitivity and 90% specificity^[16]^. Additional promising serum markers include FIB-4, KRT18, ALP (alkaline phosphatase) and bilirubin (a good indicator of more severe liver damage).

Magnetic resonance imaging (MRI) and elastrography (MRE) have been demonstrated to be highly accurate in detecting hepatic steatosis and fibrosis (for review, see^[17]^). Advanced fibrosis can also be detected through transient elastography (Fibroscan)^[18-20]^ and grade of steatosis through ultrasonography^[21]^ Xenon-133 liver scan^[22]^. As shown in a recent longitudinal study^[23]^monitoring liver fibrosis caused by viral infection or in conjunction with NAFLD/NASH, serum markers as well as imaging (e.g. Fibroscan) are well suited to predict the clinical outcome of NASH, a conclusion supported in a related study^[24]^ (for recent reviews-see^[25-26]^). It is therefore possible to consider the introduction of one or more non-invasive diagnostic modalities as part of routine diagnostics. Challenges and limitations nevertheless remain. Diagnosis and staging of NASH are difficult without biopsy and can only be inferred through fibrosis. As NAFLD and NASH overlap with the metabolic, imaging-based diagnostics is limited due to the white adipose tissue. Also, imaging instruments (e.g. MRI, MRE, Fibroscan) are expensive, require highly qualified personnel and as such, are restricted to specialized and academic centers. Development of new metabolic (bio)markers are therefore needed to complement existing ones to better stage NAFLD from NAFL through NASH with increasing fibrosis to cirrhosis.

## Current development of improved NALD/NASH biomarkers

Animal model systems exist for both NAFLD and NASH providing a homogenous genetic background and a controlled environment. A rich diet combined with fructose is often used to induce NAFLD and NASH in rodents, either in wildtype or genetically modified animals (e.g. *ob/ob* or *db/db* mice deficient in leptin production or leptin binding, respectively. Such animal model systems display many of the hallmarks of liver disease (for a recent review, see^[27]^) though with some limitations (e.g. in the induction of disease and an insufficient histopathological representation compared to human NAFLD/NASH (for review, see^[28]^). Given the limited success of pharma industry using rodent model systems in drug development, many have questioned the usefulness of rodent model systems and instead, increasingly promote the use of human material^[29-33]^. This includes the use of embryonic stem cells, induced pluripotent stem cells and adult stem cells that can either be used to generate differentiated functional cells or organoids; self-organizing multicellular structures containing multiple cell types that mimic organ structure and function. Alternatively, material can be obtained from patients and organ donors including body fluids (e.g. blood, urine), tissue and whole organs. Biomarker research using human-derived material, however, has its own limitations. Restricted availability, large variability and ranging quality of human-derived material present significant problems as do alignment of research data with patient/donor data, the latter being safeguarded to ensure patient and donor confidentiality. In our case, human research material has been obtained from donor livers and from patients undergoing liver surgery. As expected, this material displays great variability but importantly, provides snap-shots of the NAFLD/NASH disease state. Such snap-shots combine genetic and environmental factors that are difficult to mimic in an animal model system. With respect to sample variability, we found that this can be beneficial in that it has revealed new and unexpected insights and enabled hypothesis-driven research (e.g. delineating pathways in a disease context). Despite limited availability and large variability, the use of human material for NAFLD/NASH research has proven valuable (manuscript in preparation, Nilsson, Metrakos* et al.*).

With respect to alignment of research data with patient/donor data, were centlydeveloped a framework for incidental research findings such that these can get back to the physician treating the patient (if deemed significant by an independent board)^[34]^. In this framework, patient confidentiality is guaranteed through coded keys held by a third party. An important component of this framework is also informed consent. Using a similar approach, it should be possible to design legal and ethical protocols to enable research data to be more fully aligned with patient data. For this to work, emphasis needs to be put on policy development in the context of an integrated biobank framework including access and review board dealing with requests for research material and associated information. Equally important is the standardization of sample procurement, handling and long-term storage. Once in place, the prospect of finding new biomarkers will likely improve. Below follows a summary of existing markers ranging from genetic, epigenetic, protein and metabolic markers.

Genome wide association (GWAS) studies of NAFLD and NASH have already yielded genetic markers (variants). Of the more wide-spread ones, PNPLA3^I148M ^(rs738409) shows the strongest correlation with hepatic steatosis and liver fibrosis^[35-40]^. A somewhat weaker yet strong association with hepatic steatosis and liver fibrosis is also seen with TM6SF2^E167K^ (rs58542926)^[41]^, a protein associated with lipidation of secreted VLDL particles^[42]^. Even though carriers of the PNPLA3^I148M^ and the TM6SF2^E167K^ variants have higher liver fat content and increased risk of developing NASH, genotyping is not recommended routinely^[9]^. Other genetic variants include ApoCIII; FDFT1, a farnesyl transferase; NCAN, a chondroitin sulfate proteoglycan; PPP1R3B, a protein phosphatase; GCKR, a regulatory protein of glucose kinase; and LYPLAL1, an enzyme with unknown substrate specificity^[37, 43-44]^. A number of additional variants have been identified awaiting independent verifications (reviewed in^[45]^).

Epigenetic regulation and modifications are also thought to contribute to NAFLD and NASH. This includes micro RNAs where miR-122, κ-192 and κ-375 correlatewell with both NAFLD and NASH.Thesecan be used to distinguish NASH from simple steatosis with similar predictive values as KRT18, ALTor AST and in addition, offer targets for intervention^[46]^. Other types of epigenetic changes include histone modifications and DNA methylations. So far, only a few human studies have been conducted. Animal studies demonstrate changes in chromatin structure via histone modifications^[47]^, aberrant histone trimethylation of lipid catabolism and PPARα genes^[48]^, changes in DNA methylation upon depletion of methyl donors and post translational modifications of transcription factors (for reviews, see^[49-50]^).

The predictive value of the above listed markers remains uncertain as different studies show great variability in the assessment of disease state and progression (reviewed in^[49]^). As such, the cost/benefit ratio remains high precluding the use of these in (routine) diagnostics.

## Molecular pathophysiology of NAFLD and NASH

Most agree that NAFLD can be viewed as the hepatic expression of the metabolic syndrome. As NAFLD and NASH are chronic diseases, it is difficult to discern how either feeds into different co-morbidities associated with the metabolic syndrome or, how NAFLD and NASH might develop as a consequence of the metabolic syndrome. It is clear that significant overlap exists between NAFLD/NASH and obesity, pre-diabetes, hypertension, T2D as well as CVD. A long favored hypothesis explaining how NAFL progresses into NASH was the “two-hit” hypothesis whereby hepatic steatosis is the consequence of overweight/obesity (constituting the first hit). Progression of NAFL to NASH is then induced by an additional assault on the liver (e.g. lipotoxicity through circulating free fatty acids such as palmitate). This hypothesis is now considered obsolete and is replaced by the multiple hit hypothesis^[51]^. Lipotoxicity, ER stress, hormonal and cytokine secretion from adipose tissue, changes in gut microbiota, medication, genetic, epigenetic factors and insulin resistance all contribute to NAFLD progression^[51]^. For example, insulin levels in patients afflicted by T2D range from very low (due to pancreatic beta cell failure to produce insulin) to higher than normal levels (due to insulin resistance). In those with low insulin production, hormone-sensitive lipase becomes constituently activated resulting in TG breakdown and release of free fatty acids (FFAs) from adipose tissue. These are then taken up by the liver and either re-released *via* VLDL or stored in hepatic CLDs. Increased levels of circulating TGs in turn contribute to the development of CVD as most if not all VLDL secreted in conjunction with NAFLD are in the form of VLDL_1_ containing ApoC3 (ApoCIII) (for reviews, see^[52-53]^).

Loss of function polymorphism of ApoC3 results in lower risk of CVD and lower amounts of circulating TGs. The exact mechanistic reasons remain unclear although it is known that ApoC3 inhibits both lipoprotein lipase (LPL) and hepatic lipase-mediated lipolysis and that this protein facilitates VLDL_1_ assembly and secretion^[54-55]^. The activity of ApoCIII is governed by O-linked glycosylation as mutations in polypeptide N-acetylgalactosaminyltransferase 2 (GALNT2) results in a modulationof ApoCIII function decreasing its ability to inhibit LPL-mediated lipolysis of VLDL-bound TG^[56]^. If this affects hepatic steatosis and fat accumulation in other tissues remains to be tested. The ability to clear lipids via secreted VLDL particles is one of the more important routes whereby the hepatocyte can offset uptake of circulating FFAs alleviating overall steatosis. Other means are through upregulation of β-oxidation or unconventional secretion. A candidate pathway for unconventional secretion has been highlighted in differentiated 3T3-L1 adipocytes with respect to the secretion of aP2 (FABP4) which contributes to increased liver glucose secretion and consequently, hyperglycemia and T2D^[57]^. This pathway involves inclusion into multi-vesicular bodies, an endo/lysosomal compartment essential for antigen presentation. Similarly, a portion of adiponectin appears to follow the same route as a P2. Recent work also shows that some forms of autophagy are intimately coupled with unconventional secretion (reviewed in^[58]^).Whether or not this enables free fatty acids or triglycerides to be secreted remains to be determined.

The ability to clear hepatic triglycerides through an upregulation of autophagy has been demonstrated in several studies (for a recent review, see^[59]^). Caffeine ameliorates the symptoms of both NAFLD and NASH (for recent reviews, see^[60-61]^). It is estimated that a daily consumption of 2 cups of brewed regular coffee is sufficient to stimulate clearance of hepatic triglycerides through lipophagy coupled toβ-oxidation^[62-63]^. Caffeine-induced autophagy does not appear restricted to hepatic tissue and is also seen elsewhere including skeletal and neuronal tissue. Dietary lipids such as omega-3 fatty acids are also known to lower circulating triglyceride levels through decreased VLDL secretion^[64]^ coupled with induced hepatic autophagy^[65]^ and presumably, increased β-oxidation. In mice, this leads to improvement in NAFL, NASH as well as fibrosis^[66]^. Other dietary-based management strategies also exist (e.g. dietary polyphenols^[67]^) and when viewed together, offer possible avenues for treatment. At this stage, however, no clinical studies have been performed to show a clear reduction in the rate of disease progression from NAFL to NASH, fibrosis into a terminal liver disease stage.

The clearance of hepatic lipids through an upregulation of autophagy (i.e. lipophagy), increased β-oxidation, VLDL secretion and/or unconventional secretion must exceed the rate whereby neutral lipids are taken up or synthesized. This is far from simple to achieve. First, multiple lines of evidence suggest that CLDs may serve as obligatory intermediates in autophagy-related processes^[68-69]^. In other words, that CLDs form to supply the forming autophagosome with lipid material (e.g. phospholipids). Lipid content is also intimately intertwined with ER stress such that lipid composition effects the unfolded protein response (for review, see^[70]^). Also, that reactive oxygen and nitrogen species causes ER stress by modifying proteins and lipids. This, in turn, promotes CLD formation.

Another promotor of CLD formation is uric acid linking renal impairment to the development of NAFLD^[71]^. Addition of uric acid to hepatoma cells resulted in the induction of ER stress and activation of SREBP-1c, an ER bound transcription factor that upon activation, is proteolytically cleaved and translocates to the nucleus to promote transcription of genes involved in fatty acid synthesis (e.g. ACC1, FAS and SCD1). This mechanistic framework, linking ER stress to CLD formation through SREBP-1c, extends to autophagy through MTORC1 and LPIN1. During growth and high nutrient conditions, the MTORC1 complex is active inhibiting autophagy while promoting protein synthesis and the activity of transcription factors such as SREBP-1c. LPIN1, a phosphatidic acid (PA) phosphatase converting PA to diacylglycerol (DAG) becomes phosphorylated by the active MTORC1 preventing its entry into the nucleus^[72]^. This prevents LPIN1 to inhibit the nuclear activity of SREBP-1c.

LPIN1 is also required for autophagic flux through the activation of the PKD-VPS34 signaling pathway^[73]^. Again, its ability to convert DAG to PA is inhibited by MTORC1-dependent phosphorylation ensuring multiple blocks of autophagy during growth and high nutrient conditions. Activation of MTORC1, however, is also linked to ER stress causing conflicting signaling cascades, on the one hand promoting lipogenesis and on the other hand, preventing autophagy and CLD clearance through the inactivation of LPIN1 (see for example, work in Zebra fish^[74]^). Such dysregulation might have a direct relevance to the progression of NAFLD and offers direct avenues for intervention through drugs that inhibit MTORC1 (e.g. rapamycin derivatives).

With respect to T2D and pre-states of T2D, the hallmarks of liver insulin resistance are unabated gluconeogenesis and unsuppressed lipogenesis. Gluconeogenesis and lipogenesis are respectively regulated through the insulin/IRS2 and the insulin/IRS1 pathways. Under normal conditions, insulin action through the IRS2 pathway leads to phosphorylation and nuclear exclusion of FOXO1 with dampened expression of gluconeogenesis genes such as G6Pase and *PEPCK* but also MTP and apoC-III that are both essential for VLDL_1_ assembly. Sustained hyperinsulinemia and hyperglycemia with compromised insulin/IRS2/FOXO1 signaling yields high expression of MTP and apoC-III with an overproduction of TG-VLDL_1_^[52, 75-79]^ yet causes of compromised insulin/IRS2/FOXO1 signaling remain unclear. It is also possible that hepatic accumulation of CLDs directly impacts glucose intolerance and insulin resistance.

## Disease management and possible treatment of patients with NAFLD

Lifestyle modifications including dietary changes, restricted calorie intake and a minimum of weekly exercise have been shown to improve NAFL as deduced by ultrasound or MRI (summarized in^[16]^) as well as NASH^[80]^.Changes in lifestyle is therefore recommended to reduce NAFL and to improve NASH^[16]^. Use of metformin show only limited improvements in NAFL but has no significant impact on liver histology and is therefore not recommended in the treatment of adults with NASH^[16]^. In contrast, pioglitazones have been shown to improve NASH but should be restricted to non-diabetic patients. Also, long-term safety of pioglitazones is under considerable debate and is either not recommended or severely restricted in use. This is due to increased risks in coronary events^[81]^. Vitamin E administered daily improves liver histology in non-diabetic patients with NASH and is recommended as a first line treatment^[16]^ and other dietary supplements can be considered including those mentioned above though at present, may be premature awaiting clinical studies.

## Concluding remarks

The metabolic dysregulation leading to NAFLD and NASH and consequent dysregulations caused by NAFLD and NASH impacting other disease states (e.g. T2D and CVD) are progressive in nature each feeding into the other. As NAFLD and NASH are increasing in prevalence and already affecting large part of the population, it is important to understand that the consequences of NAFLD and NASH will place a huge burden on healthcare. A first step should therefore be to educate primary care physicians to recognize risk factors associated with NAFLD and in particular, NASH with fibrosis, such that the appropriate investigation can be carried out. A second step should be to implement serum-based diagnostics and imaging modalities as part of routine diagnostics. It should be stated that it is presently difficult to design prospective studies; to recruit relevant cohorts that are representative of the population. This difficulty is compounded by granting agencies still questioning the relevance of NAFLD as a metabolic disease despite its documented impact, prevalence and increased mortality rate (in the case of NASH, fibrosis and cirrhosis). Large NAFLD and NASH consortia have nevertheless been established both in the US and the EU that all focus on a better understanding of disease, improved diagnostics and enhanced treatment strategies.
